# The impact of strict lockdowns on the mental health and well-being of people living in Australia during the first year of the COVID-19 pandemic

**DOI:** 10.1192/bjo.2023.65

**Published:** 2023-05-24

**Authors:** Anita M. Y. Goh, Christa Dang, Rushani Wijesuriya, Karen E. Lamb, Maya G. Panisset, Pragya Gartoulla, Esther Tan, Frances Batchelor, Bianca Brijnath, Briony Dow

**Affiliations:** Faculty of Medicine, Dentistry and Health Sciences, The University of Melbourne, Australia; and Aged Care Division, National Ageing Research Institute, Melbourne, Australia; Clinical Gerontology Division, National Ageing Research Institute, Melbourne, Australia; Methods and Implementation Support for Clinical and Health Research Hub, Faculty of Medicine, Dentistry and Health Sciences, The University of Melbourne, Australia; and Clinical Epidemiology and Biostatistics Unit, Murdoch Children's Research Institute, Melbourne, Australia; Methods and Implementation Support for Clinical and Health Research Hub, Faculty of Medicine, Dentistry and Health Sciences, The University of Melbourne, Australia; and Centre for Epidemiology and Biostatistics, Melbourne School of Population and Global Health, The University of Melbourne, Australia; Faculty of Medicine, Dentistry and Health Sciences, The University of Melbourne, Australia; and Clinical Gerontology Division, National Ageing Research Institute, Melbourne, Australia; Social Gerontology Division, National Ageing Research Institute, Melbourne, Australia; Aged Care Division, National Ageing Research Institute, Melbourne, Australia; Social Gerontology Division, National Ageing Research Institute, Australia; and School of Social Sciences, University of Western Australia, Australia; Faculty of Medicine, Dentistry and Health Sciences, The University of Melbourne, Australia; and Director, National Ageing Research Institute, Melbourne, Australia

**Keywords:** COVID-19, mental health, depression, disaster, anxiety

## Abstract

**Background:**

There are limited longitudinal studies on the effects of the COVID-19 pandemic on mental health and well-being, including the effects of imposed restrictions and lockdowns.

**Aims:**

This study investigates how living in a pandemic, and related lockdowns and restrictions, affected the mental health of people living in Australia during the first year of the COVID-19 pandemic.

**Method:**

A total of 875 people living in Australia participated in a longitudinal survey from 27 May to 14 December 2020. This time period includes dates that span pre-, during and post-wave 2 lockdowns in Australia, with strict and sustained public health measures. Linear mixed models were fitted to investigate the effect of lockdown on depression and anxiety symptoms.

**Results:**

Symptoms of depression and anxiety improved over time, during and after lockdowns. More adverse mental health symptoms were observed for people with a history of medical or mental health problems, caring responsibilities, more neurotic personality traits or less conscientiousness, and for people who were younger. People who reported being more conscientious reported better mental health.

**Conclusions:**

Despite notoriously strict lockdowns, participants did not experience a deterioration of mental health over time. Results suggest a lack of significant adverse effects of lockdown restrictions on mental health and well-being. Findings highlight cohorts that could benefit from targeted mental health support and interventions, so that public policy can be better equipped to support them, particularly if future strict public health measures such as lockdowns are being considered or implemented for the COVID-19 pandemic and other disasters.

The potential for the COVID-19 pandemic to affect mental health and well-being was recognised early on in the pandemic, as a result of concerns around contracting the virus itself as well as from the measures used to contain its spread.^1^ Unprecedented public health measures and restrictions were globally imposed, including working from home mandates, childcare and school closures, restrictions on travel and activities, and physical distancing/quarantine/isolation measures for significant periods of time. These occurred in the context of fear and anxiety about how the COVID-19 virus might affect individuals, communities, healthcare services and society. Although public health restriction measures were necessary to ‘stop the spread’, particularly in the absence of vaccines in the early stages of the pandemic, there have been psychosocial (and economic) costs as a result.^2^

It is crucial to understand how the virus itself and associated restrictions affected the short- and long-term mental health and well-being of people, and how these factors track over time. For these reasons, an international call to action was made for high-quality data on the mental health effects of the COVID-19 pandemic.^3^ Studies have reported increased levels of psychological distress and symptoms of depression and anxiety compared with pre-pandemic levels.^4–8^ COVID-19 restrictions have also affected social connectedness, with reports of increased social isolation and loneliness associated with increased psychological distress.^6,9^ Other downstream effects of public health measures, including job losses and reduced financial resources, have also adversely affected mental health.^8,10,11^ Furthermore, mandatory contact tracing and quarantine have contributed to feelings of anxiety and guilt about the effects of infection, quarantine and stigma.^12^ COVID-19 infections and associated restrictions have also shown to contribute to, or exacerbate, existing chronic mental illness, including anxiety, depression, post-traumatic stress disorder and substance misuse.^13^

However, the majority of studies examining the mental health and well-being effects of the COVID-19 pandemic in Australia are based on cross-sectional data collected in the early days of the pandemic, when public health orders were not as strict and threat of community transmission was lower (i.e. the first wave). Those studies, which reported that mental health and well-being was substantively lower than pre-pandemic normative estimates, may have captured the immediate, acute shock of a global pandemic following a severe bushfire season.^4,5,7,8^ There are limited longitudinal mental health and well-being studies about people living in Australia during the pandemic, and only one of these reported data collected before the second wave.^10,11,14,15^ This study reports results of one of the few surveys in Australia that capture data leading into and following the second wave, and the infamously strict restrictions imposed during that time.

## Australia during the first year of the COVID-19 pandemic

In Australia, widespread restrictions of movement, distancing measures and physical isolation, or ‘lockdowns’, were implemented from March 2020 as a response to Australia's first locally transmitted case of COVID-19. Up until October 2021, Australia had three major ‘COVID-19 waves’ or distinct peaks of infections that led to widespread lockdowns. The general dates are: wave 1 in March and April 2020 (affecting all states and territories with most infections acquired overseas), wave 2 from end of June to October 2020 (mainly affecting the state of Victoria with most infections acquired via community transmission)^16^ and wave 3 from August 2021 until October 2021 (from the Delta variant of COVID-19).^17^ In 2020, there were 899 deaths from COVID-19 registered in Australia, and 89% of deaths were in Victoria (*n* = 800).^16^

Most Australians experienced stay-at-home orders during wave 1 of COVID-19 infections. These first wave restrictions were easing in May 2020 (when our survey began collecting data), with single-digit daily case numbers in Victoria at the start of June 2020. However, case numbers were soon to rise as a result of hotel quarantine breaches, resulting in the second wave. The state of Victoria subsequently experienced a large and prolonged second wave of COVID-19 infections, which resulted in approximately 19 000 recorded cases and 800 deaths, linked primarily to 222 outbreaks in residential aged care homes.^16,18^ The government's strict lockdown strategy was effective in eliminating community transmissions,^19^ and restrictions were eased in Victoria from 26 October 2020, when zero community transmissions were recorded in all Australian states. There was an extended period in Australia with no community transmissions until the third wave began. When the last lockdown in Victoria ended on 21 October 2021, the people of Melbourne (a city of 5 million people) had spent a cumulative 262 days under strict lockdown conditions since March 2020, one of the most stringent in the world, leading to the nickname ‘world's most locked down city’.^20^ These lockdowns were characterised by long periods of border closures, imposed curfew, childcare and school shutdowns, closure of retail and hospitality, restrictions on movement (≤5 km from home) and restrictions of visitors to homes, with only four essential reasons to leave home (shopping for essential goods or services, work or study if not possible from home, seeking or giving care, and exercise for limited times). Getting a COVID-19 vaccination was only added as a fifth reason to leave home in April 2021, when the first vaccines were available.

We conducted a longitudinal survey investigating the impact of the COVID-19 pandemic on the mental health and well-being of people living in Australia during the first year of the pandemic, from 27 May 2020 to 14 December 2020. The survey was modelled on the UCL COVID-19 social study, and had the broad aim of exploring the emotional, mental, health and societal behaviours and experiences of people during the COVID-19 pandemic.^21,22^ Our analysis of baseline cross-sectional data from this survey,^23^ collected from 803 participants during the peak of the second wave (from 27 May to 19 August 2020), revealed that self-rated knowledge about COVID-19 was high, adherence to government recommendations to prevent the spread of COVID-19 was high and trust in the Australian government's handling of the COVID-19 pandemic was high, as were participants’ confidence in the Australian healthcare system during the COVID-19 pandemic. Participants also strongly believed that access to essentials (such as food, water and medicines) would be maintained.

In the current study, the primary aim was to investigate the impact of the pandemic and major lockdowns on the mental health and well-being of people residing in Australia across three periods: pre-major lockdown, during major lockdown and post-major lockdown in 2020. As Victoria experienced a prolonged lockdown period in 2020, secondary analyses assessed if changes in depression or anxiety differed by participant location (metropolitan Melbourne, regional Victoria or outside of Victoria), or according to baseline participant characteristics. We hypothesised that greater depression and anxiety symptoms would be observed during the major lockdown period compared with pre- and post-major lockdown. We also hypothesised that people living in areas with longer and more restrictive lockdowns (i.e. Victorians) would experience poorer mental health.

## Method

### Study population and participant recruitment

Participant recruitment was conducted by the National Ageing Research Institute, using a convenience sampling approach through a variety of channels, including media, social media, targeted advertising, and personal and professional networks. Participation was open to any person living in Australia during the survey, aged 18 years or above. Because the pandemic had population-wide effects that were occurring in real time, and because of the time-sensitive nature of this research, a convenience sampling approach was used to enable quick collection of data at a crucial time during the pandemic, and to generate a large sample in a short period of time.

Data collection began on 27 May 2020 and continued until 14 December 2020 via an online platform (REDCap; Vanderbilt University, Nashville, TN, USA; https://projectredcap.org/), or via hard-copy paper versions, which could be posted to participants with a postage-paid reply envelope. New participants could join the study and complete the baseline assessment at any point during that period. The survey collected sociodemographic information, a basic health profile, occupational and social roles (e.g. key worker or carer status), and information about living situation. The baseline questionnaire required approximately 20 min to complete and weekly follow-up questionnaires took approximately 15 min, where participants shared ongoing information about their mental health, loneliness, stressors, adherence to restrictions, confidence in government management, and exercise and social behaviours. Study participants were also invited to participate in a single qualitative interview, and findings will be reported separately.

The study did not aim to be representative of the Australian population, but instead aimed to have representation across all major sociodemographic groups. The study received ethical approval from the University of Melbourne (approval number: 2056799.1).

#### Participants

**Fig. 1:**
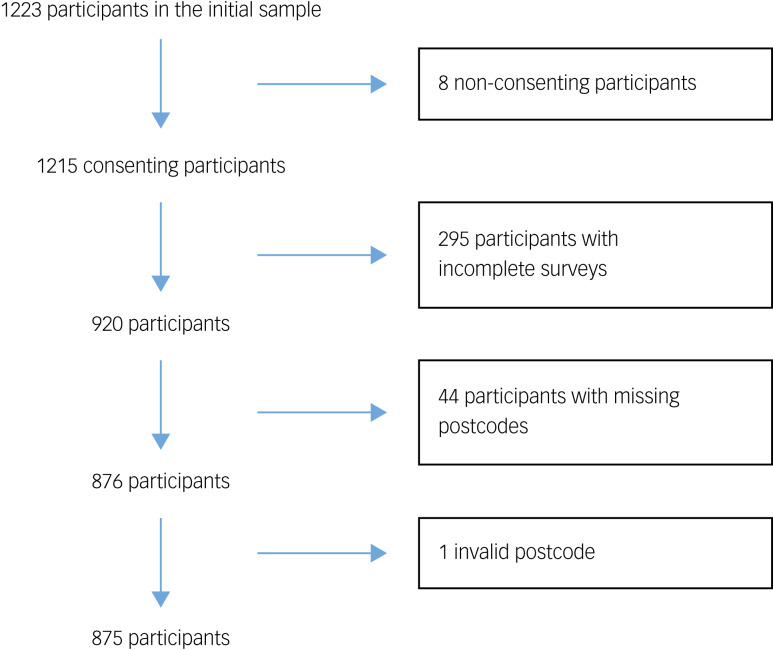
Participant flow chart for the eligible sample.

A total of 1223 individuals began the online survey, with 1215 (99.3%) providing written consent to be included in the study. Among those that provided consent, 295 (24.3%) had incomplete surveys; these individuals did not provide data on the outcome measures or for several key covariates of interest required for the analyses (see Supplementary Table 1 available at https://doi.org/10.1192/bjo.2023.65). Of those with complete surveys (*n* = 920), 45 individuals had missing or invalid postcode information (see Supplementary Table 2). Excluding individuals with incomplete surveys and incomplete/invalid postcodes resulted in a sample size of 875 participants eligible for analyses. See Fig. 1 for the participant flowchart.

### Measures

#### Outcome: mental health

Self-reported mental health was assessed at all time points, using two outcomes: depressive symptoms measured with the nine-item Patient Health Questionnaire (PHQ-9)^24^ and anxiety symptoms measured with the seven-item Generalised Anxiety Disorder (GAD-7)^25^ tool. These tools align closely with diagnostic criteria for major depressive disorder and generalised anxiety disorder, respectively.^24^ In both tools, items ask how often symptoms were bothering respondents in the past week. PHQ-9 scores range from 0 to 27, with scores of 5, 10, 15 and 20 representing cut-off points for mild, moderate, moderately severe and severe depression, respectively. GAD-7 scores range from 0 to 21, and scores of 5, 10 and 15 represent cut-off points for mild, moderate and severe anxiety, respectively.

#### Outcome: personality traits

The Short 15-item Big Five Inventory (BFI-S)^26^ was completed by participants once, at baseline. This measures five key dimensions of personality: openness, conscientiousness, extroversion, agreeableness and neuroticism. Scores for each of the five personality dimensions were determined from the mean score across the three items associated with each dimension. Higher scores for each personality trait represent greater strength for that trait.

#### Exposure: lockdown period

Victoria was the only Australian state/territory that entered a prolonged major lockdown in 2020. Metropolitan Victoria (i.e. Melbourne) entered major lockdown at a different time to the rest of Victoria. Participant responses were classified as pre-, during and post-major lockdown, according to both the date the survey was completed and the participant postcode of residence.

The pre-major lockdown period was defined as between 27 May (study initiated) and 1–9 July 2020 (for Melbourne), and between 27 May (study initiated) and 2 August 2020 (for regional Victoria). The lockdown period was defined as between 1–9 July and 27 October 2020 (for Melbourne), and between 3 August and 27 October 2020 (for regional Victoria). The post-major lockdown period was defined as between 28 October and 14 December 2020 (study end).

There was variation in the metropolitan Victoria lockdown as some hot-spot postcodes were placed in lockdown before others (further details are in Supplementary Table 3). Participants living outside of Victoria were considered as a comparator group. For the comparator group, the pre-major lockdown period was considered to be the period before the whole of Victoria being in major lockdown (i.e. 27 May to 2 August 2020).

#### Covariates

Baseline covariates considered to be potentially associated with mental health outcomes included age (years); gender identification (man, woman, other/prefer not to say); country of birth (Australia, not Australia); caring responsibilities for older relatives or friends, or for people with long-term conditions or disabilities (yes, no); history of clinically diagnosed mental health conditions (yes, no); currently engaged in paid employment, including casual, part-time, full-time or self-employment (yes, no); and area of residence (metropolitan Victoria/Melbourne, regional Victoria, not Victoria). These covariates were selected based on well-known factors considered to be potentially associated with mental health outcomes in the literature.^7,8,10,11,15,27–29^ Country of birth was included as an exploratory variable to investigate whether separation from family owing to COVID-19-related travel restrictions affected mental health. Area of residence was included as lockdowns varied according to location.

Area-level socioeconomic status was derived by linking participant postcodes to the 2016 Australian Bureau of Statistics Socioeconomic Index For Areas (SEIFA) Index of Relative Socio-economic Advantage and Disadvantage (IRSAD).^30^ This index ranks areas in Australia according to relative socioeconomic advantage and disadvantage based on information from the 5-yearly Census of Population and Housing. SEIFA quintiles were considered ranging from 1 (most disadvantaged) to 5 (least disadvantaged).

Secondary analysis within the Victorian subsample examined which of the following time-fixed risk factors and personality traits were associated with the change in mental health over time across the three time periods: pre-major lockdown, during major lockdown and post-major lockdown in Victoria: (a) increased risk because of older age (≥65 years with conditions, or ≥70 years; yes/no); (b) caring responsibilities (yes/no); (c) history of mental health conditions (yes/no); (d) medical history/comorbidities (excluding pregnancy; yes/no); (e) country of birth (born in Australia/born outside Australia); (f) essential/key worker (i.e. health, social care or relevant related support, teacher or childcare, transport, food chain work, key public service, local or national government work delivering essential public service, utility work, medicine or protective equipment production or distribution; yes/no); (g) having one or more children in the household (yes/no); and (h) personality traits as measured by the BIF-S, i.e. extroversion, agreeableness, openness, conscientiousness and neuroticism.

Participants were considered at increased risk of serious COVID-19 outcomes if they were aged 65 years and above with a medical condition or aged above 70 years, as per the Department of Health's definition.^31^ Participants were defined as having a medical history of comorbidities if they reported having high blood pressure, diabetes, heart disease, lung disease, cancer or other chronic physical health condition or disability.

### Statistical analysis

Using Stata version 15.1 for Windows, descriptive statistics for the baseline participant characteristics (*n* = 875) were calculated along with missing data proportions (Table 1). Linear mixed models (LMMs) were fitted to investigate the effect of lockdowns on mental health (depression and anxiety symptoms), with random intercepts to account for within-individual correlations. The results from LMMs provided estimates of the mean differences, with corresponding 95% confidence intervals and *P*-values, in total severity scores comparing the during and post-major lockdown period with the pre-major lockdown period. Unadjusted models included only the lockdown period; adjusted models included age, gender, country of birth, caring responsibilities, previous mental health conditions, paid employment, area of residence and area-level socioeconomic status.

**Table 1:** Descriptive characteristics of the eligible study sample at the point at which they joined the study (*n* = 875)

		*N* = 875
Characteristics		*n* (%)
Age, mean (s.d.)	Years	59.4 (16.5) (*n* = 869)
Identified gender	Woman	693 (79.2%)
	Man	165 (18.9%)
	Other/prefer not to say	5 (0.6%)
	Missing	12 (1.4%)
Employment status	No	393 (44.9%)
	Yes	476 (54.4%)
	Missing	6 (0.7%)
Carer responsibilities	No	660 (75.4%)
	Yes	183 (20.9%)
	Missing	32 (3.7%)
History of clinically diagnosed mental health problems	No	688 (78.6%)
	Yes	172 (19.7%)
	Missing	15 (1.7%)
Area of residence affected by the metropolitan Victoria lockdown	Metropolitan Victoria	547 (62.5%)
	Non-Victoria	206 (23.5%)
	Regional Victoria	122 (13.9%)
SEIFA IRSAD quintile	Quintile 1 (most disadvantaged)	62 (7.1%)
	Quintile 2	76 (8.7%)
	Quintile 3	131 (15.0%)
	Quintile 4	202 (23.1%)
	Quintile 5 (least disadvantaged)	404 (46.2%)
Born in or outside Australia	Born in Australia	631 (72.1%)
	Born outside Australia	241 (27.5%)
	Missing	3 (0.3%)

SEIFA, Socio-Economic Indexes for Areas; IRSAD, Index of Relative Socio-economic Advantage and Disadvantage.

To determine if change in depression or anxiety differed by participant location (metropolitan Victoria/Melbourne, regional Victoria or outside of Victoria), an interaction between area of residence and lockdown period was included in adjusted LMMs. In secondary analyses, an interaction between each potential time-fixed baseline risk factor and lockdown period was included in adjusted LMMs, to assess if these factors were associated with differences in depression or anxiety symptoms over time for Victorian participants. The eight factors listed above were considered in separate LMMs in this exploratory analysis.

A complete-case analysis was conducted, omitting participants with missing data for any of the covariates (*n* = 53), as well as any participants who had missing outcome values at all time points for which they provided data (*n* = 55 for the PHQ-9; *n* = 58 for the GAD-7). This resulted in a complete-case sample size of 767 (87.7% of the 875 eligible participants) for the PHQ-9 and 764 (87.4%) for the GAD-7. Descriptive characteristics of the full sample, complete-case sample and omitted participants were considered to determine if characteristics were comparable between those who were and were not included in the analysis (see Supplementary Tables 4 and 5). Descriptive characteristics for the complete-case sample and omitted sample of participants were compared to determine if there were any differences in the characteristics of included and excluded participants (Supplementary Table 4). The two samples were generally comparable, although there was a slightly higher percentage of participants who were female (81% *v*. 74%), employed (56% *v*. 48%) or had no caring responsibilities (79% *v*. 74%) in the complete-case sample compared with the omitted sample of participants.

## Results

Characteristics of the eligible study sample at baseline are shown in Table 1. Mean participant age was 59.4 years and the majority identified as women, were born in Australia and lived in Melbourne, Victoria. Most participants also resided in the most advantaged SEIFA IRSAD quintile categories, had no caring responsibilities and reported no previous clinically diagnosed mental health problems. Employment status was more balanced, at 55.4% employed. Of the complete-case samples for the PHQ-9 (*n* = 767) and GAD-7 (*n* = 764), 668 (approximately 87%) provided longitudinal data. Each participant on average provided data for 9.7 (s.d. = 7.4) and 9.8 (s.d. = 7.1) weeks (maximum 25) for the PHQ-9 and GAD-7, respectively.

### Depressive symptoms at each time period

Mean pre-lockdown PHQ-9 score was higher than both the mean scores during and post-lockdown, indicating more depressive symptoms before lockdown (Tables 2 and 3). The estimated mean differences in PHQ-9 from the pre-lockdown to lockdown period and from the pre-lockdown to post-lockdown period, after adjusting for the potential confounders, were −0.49 (95% CI −0.69 to −0.29) and −1.35 (95% CI −1.59 to −1.12), respectively.

**Table 2:** Mean outcome measures during each time period (pre-, during- and post-lockdown)

	Pre-lockdown (*n* = 406)		During lockdown (*n* = 770)		Post-lockdown (*n* = 323)	
	*n*^a^	Mean (s.d.)^b^	*n*^a^	Mean (s.d.)^b^	*n*^a^	Mean (s.d.)^b^
Patient Health Questionnaire-9	374	6.26 (5.24)	724	5.74 (5.07)	321	3.83 (4.54)
Generalised Anxiety Disorder-7	373	4.87 (4.75)	721	4.34 (4.48)	319	3.06 (3.81)

a. Number at each time period does not equal the total number because some participants had only responded once in the survey and had missing outcome values.

b. Multiple outcomes from an individual were collapsed to individual mean before the mean across all individuals was calculated.

**Table 3:** Unadjusted and adjusted estimates of the difference in mean Patient Health Questionnaire-9 score between lockdown periods from linear mixed models (*n* = 767)

Lockdown period	Unadjusted			Adjusted^a^		
	Coefficient	95% CI	*P*-value	Coefficient	95% CI	*P*-value
Pre-lockdown	Reference			Reference		
Lockdown	−0.52	(−0.72 to −0.32)	<0.001	−0.49	(−0.69 to −0.29)	<0.001
Post-lockdown	−1.39	(−1.63 to −1.16)	<0.001	−1.35	(−1.59 to −1.12)	<0.001

a Adjusted for participant age, gender, socioeconomic status (measured by the Socio-Economic Indexes for Areas, Index of Relative Socio-economic Advantage and Disadvantage), caring responsibilities, history of previous mental health problems, born in or outside Australia and whether engaged in paid employment.

### Anxiety symptoms at each time period

The mean pre-lockdown GAD-7 score was higher than both the mean score during and post-lockdown, indicating more anxiety symptoms before lockdown (Tables 2 and 4). The estimated mean differences in GAD-7 from the pre-lockdown to lockdown period and from the pre-lockdown period to post-lockdown period, after adjusting for potential confounders, were –0.35 (95% CI −0.54 to −0.16) and −0.88 (95% CI −1.10 to −0.66), respectively.

**Table 4:** Unadjusted and adjusted estimates of the difference in mean Generalised Anxiety Disorder-7 between lockdown periods from linear mixed models (*n* = 764)

Lockdown period	Unadjusted			Adjusted^a^		
	Coefficient	95% CI	*P*-value	Coefficient	95% CI	*P*-value
Pre-lockdown	Reference			Reference		
Lockdown	−0.38	(−0.57 to −0.19)	<0.001	−0.35	(−0.54 to −0.16)	<0.001
Post-lockdown	−0.92	(−1.14 to −0.70)	<0.001	−0.88	(−1.10 to −0.66)	<0.001

a. Adjusted for participant age, gender, socioeconomic status (measured by the Socio-Economic Indexes for Areas, Index of Relative Socio-economic Advantage and Disadvantage), caring responsibilities, history of previous mental health problems, born in or outside Australia and whether engaged in paid employment.

### Change in mental health over time, depending on where a person lived in Australia

**Fig. 2:**
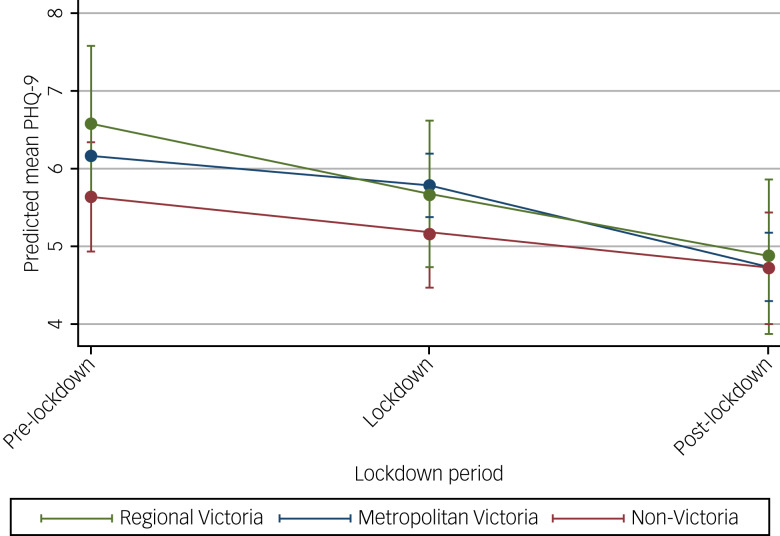
Estimated average mean PHQ-9 scores and 95% confidence intervals at pre-, during and post-lockdown by area of residence from linear mixed models.

**Fig. 3:**
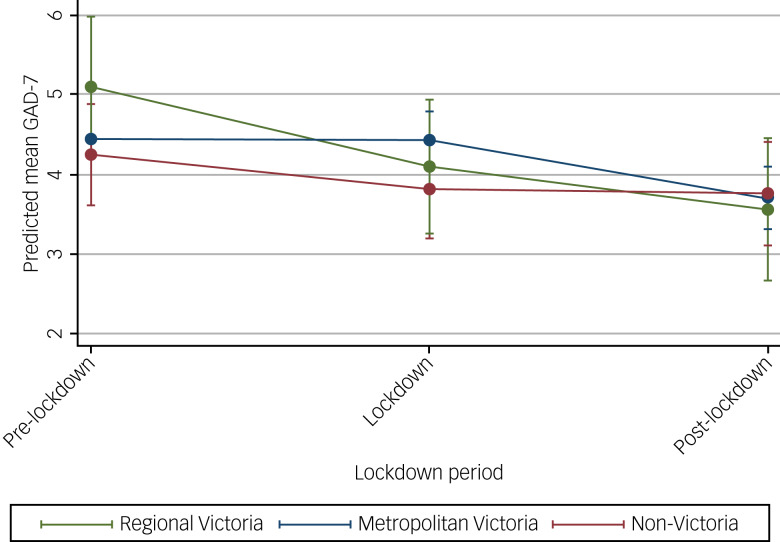
Estimated average mean GAD-7 scores and 95% confidence intervals at pre-, during and post-lockdown by area of residence.

Examination of the changes in mental health over time by participant location in Australia (Figs 2 and 3) showed no interaction effects. As in prior analyses, the results indicated symptoms of depression improved over time for all subgroups (Fig. 2), as did anxiety symptoms (Fig. 3). Full modelling results from the LMMs are presented in Supplementary Tables 6 and 7.

### Secondary analyses in the Victorian subsample

A large proportion of respondents from the sample (*n* = 669; 87%) were living in Victoria and experienced strict lockdown measures in 2020. Descriptive statistics are shown in Table 5.

**Table 5:** Descriptive characteristics of the eligible Victorian study sample when they joined the study (*n* = 669)

	Full sample *N* = 669,
	*n* (%) or mean (s.d.)
Age, years, mean (s.d.)		59.5 (17.1) (*n* = 666)
Older aged (≥65 years with conditions or ≥70 years)	Yes	263 (39.3%)
	No	403 (60.2%)
	Missing	3 (0.4%)
Identified gender	Woman	527 (78.8%)
	Man	130 (19.4%)
	Other/prefer not to say	4 (0.6%)
	Missing	8 (1.2%)
Employment status	No	308 (46.0%)
	Yes	358 (53.5%)
	Missing	3 (0.4%)
Key worker	Yes	148 (22.1%)
	No	329 (49.2%)
	Missing	192 (28.7%)
Carer responsibilities	No	520 (77.7%)
	Yes	123 (18.4%)
	Missing	26 (3.9%)
Children in household	Yes	111 (16.6%)
	No	533 (79.7%)
	Missing	25 (3.7%)
History of clinically diagnosed mental health problems	No	536 (80.1%)
	Yes	122 (18.2%)
	Missing	11 (1.6%)
Pre-existing medical conditions	Yes	413 (61.7%)
	No	245 (36.6%)
	Missing	11 (1.6%)
SEIFA IRSAD quintile	Quintile 1 (most disadvantaged)	38 (5.7%)
	Quintile 2	52 (7.8%)
	Quintile 3	102 (15.2%)
	Quintile 4	160 (23.9%)
	Quintile 5 (least disadvantaged)	317 (47.4%)
Born in or outside Australia	Australia	496 (74.1%)
	Other	172 (25.7%)
	Missing	1 (0.1%)
Personality trait, mean (s.d.)		
Extroversion		13.1 (4.1) (*n* = 631)
Agreeableness		15.6 (2.9) (*n* = 634)
Openness to experience		15.7 (3.2) (*n* = 634)
Conscientiousness		16.0 (3.0) (*n* = 628)
Neuroticism		10.9 (4.1) (*n* = 630)

SEIFA, Socio-Economic Indexes for Areas; IRSAD, Index of Relative Socio-economic Advantage and Disadvantage.

### Factors affecting depressive symptoms in the Victorian subsample

**Fig. 4:**
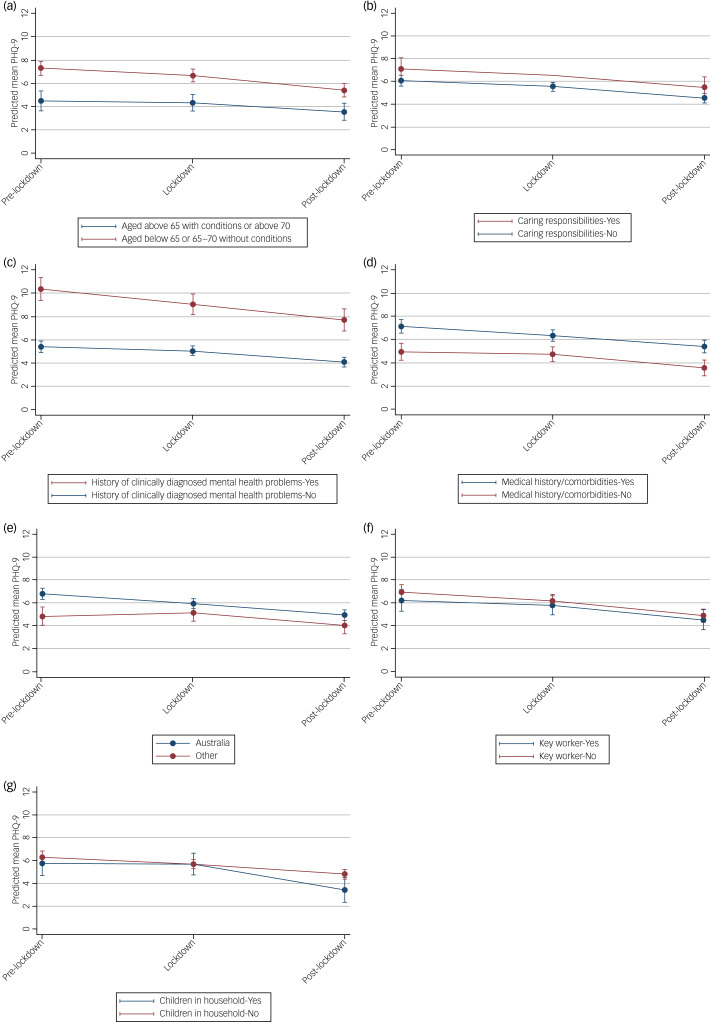
Estimated average mean PHQ-9 scores and 95% confidence intervals at pre-, during and post-lockdown, by (a) age group, (b) caring responsibilities, (c) history of clinically diagnosed mental health problems, (d) medical history/comorbidities, (e) country of birth, (f) key worker status and (g) children in the household, from linear mixed models adjusted for potential confounders.

Average PHQ-9 scores were markedly higher for those with a history of medical conditions, and for those with a history of clinically diagnosed mental health problems (at all time periods), compared with those who had no such history (Fig. 4(c, d)). PHQ-9 scores were also slightly higher for younger participants (i.e. aged below 65 or 65–70 with no medical conditions) compared with older participants.

However, as in the primary analysis, all groups show that depressive symptoms on average decreased slightly over time (Fig. 4(a–g)). There were no differences in the trend over time periods by any potential hypothesised modifying factors, indicating that the effect of lockdowns on depressive symptoms remains largely unmodified by these risk factors. For all other risk factors (caring responsibilities, country of birth, type of job, children in the household), the differences in mean PHQ-9 scores at different time points for the subgroups were small and likely not clinically meaningful, with considerable overlap in confidence intervals of the mean PHQ-9 estimates at each time point. See Supplementary Table 8 for model parameters.

### Factors affecting anxiety symptoms in the Victorian subsample

**Fig. 5:**
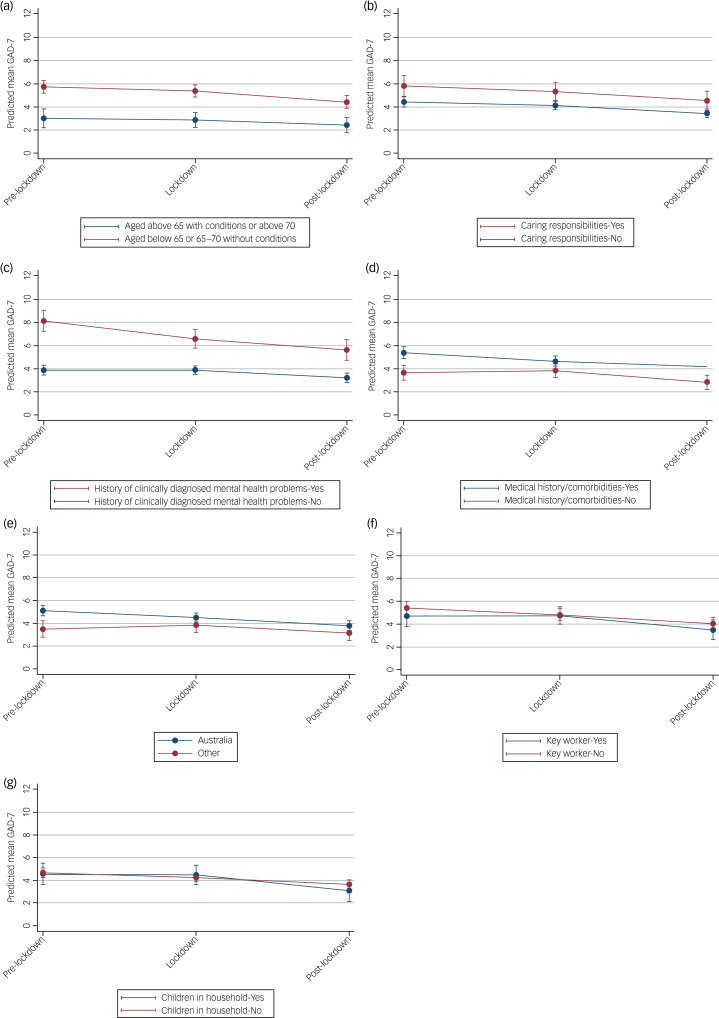
Estimated average mean GAD-7 scores and 95% confidence intervals at pre-, during and post-lockdown, by (a) age group, (b) caring responsibilities, (c) history of clinically diagnosed mental health problems, (d) medical history/comorbidities, (e) country of birth, (f) key worker status and (g) children in the household (yes/no), from linear mixed models adjusted for potential confounders.

Similar to that found for depressive symptoms, GAD-7 scores on average were higher for (a) younger people (aged below 65 or 65–70 with no medical conditions) across all time periods compared with those who were older (Fig. 5(a)), (b) for those with a history of clinically diagnosed mental health problems compared with those who had no such history (Fig. 5(c)) and (c) for those with a history of medical conditions compared with those without (Fig. 5(d)). In this case, those with caring responsibilities had higher average GAD-7 scores compared with those with no caring responsibilities (Fig. 5(b)).

As with depression symptoms, there was no interaction effect for any of the potential moderators considered. For all groups, on average, the level of anxiety decreased, indicating that anxiety symptoms improved modestly in this sample over time (Fig. 5(a–g)). For all other risk factors (country of birth, type of job, children in the household), the differences in mean scores at different time points for the subgroups are small and likely not clinically meaningful, with considerable overlap in confidence intervals of the mean GAD-7 estimates at each time point (Fig. 5). See Supplementary Table 8 for model parameters.

### Influence of personality traits on mental health outcomes

**Fig. 6:**
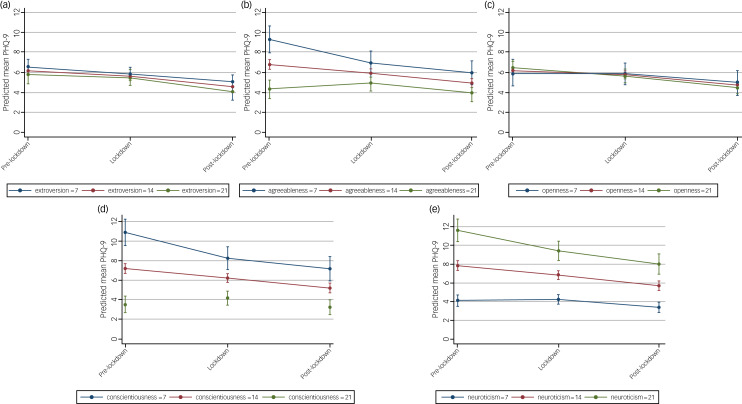
Estimated average mean PHQ-9 scores and 95% confidence intervals at pre-, during and post-lockdown, by personality traits of (a) extroversion, (b) agreeableness, (c) openness, (d) conscientiousness and (e) neuroticism, from linear mixed models adjusted for potential confounders.

**Fig. 7:**
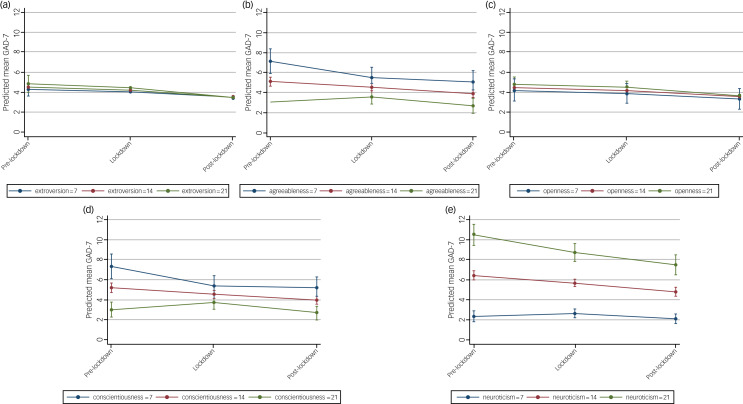
Estimated average mean GAD-7 scores and 95% confidence intervals at pre-, during and post-lockdown, by personality traits of (a) extroversion, (b) agreeableness, (c) openness, (d) conscientiousness and (e) neuroticism, from linear mixed models adjusted for potential confounders.

The effect of lockdown on symptoms of depression and anxiety were largely unmodified by personality traits (Figs 6 and 7, Supplementary Table 9). Each personality trait score was considered as a continuous covariate in LMMs, with cut-off points used in figures for illustrative purposes.

#### Depression

There was no interaction effect by personality trait (Fig. 6). However, on average, PHQ-9 scores were higher (indicating more depressive symptoms) for those with high scores for neuroticism (Fig. 6(e)), whereas on average, PHQ-9 scores were lower (indicating lower levels of depressive symptoms) for those with high conscientiousness (Fig. 6(d)). The differences in mean PHQ-9 scores at different time points for all other personality traits (extroversion, agreeableness, openness) were small, with considerable overlap in the confidence intervals of the mean PHQ-9 estimates at each time point (Fig. 6(a–c)).

#### Anxiety

As with depression, the effect of lockdowns on anxiety was unmodified by personality traits (Fig. 7). Similar to what was observed for PHQ-9, on average, GAD-7 scores were higher for those with higher scores for neuroticism (Fig. 7(e)) and lower for those with higher scores for conscientiousness (Fig. 7(d)), irrespective of time period. Again, the differences in mean GAD-7 scores at different time points for all other personality traits (extroversion, agreeableness, openness) were small, with considerable overlap in the confidence intervals of the mean GAD-7 estimates at each time point (Fig. 7(a–c)).

## Discussion

This study investigated the impact of the COVID-19 pandemic and related lockdown measures on the mental health and well-being of people living in Australia across three broad periods: pre-major lockdown, during major lockdown and post-major lockdown during the first year of the pandemic in Australia. Results showed that participants’ mental health improved throughout lockdown restrictions, with worse mental health symptoms reported before lockdown occurred. We also found that people living in areas with longer and more restrictive lockdowns (i.e. Victorians) did not experience poorer mental health as a result of the lockdown. Regardless of where participants lived (in metropolitan Victoria, non-Victoria or regional Victoria), results indicated symptoms of depression and anxiety improved over time and during and post-lockdowns. However, the change in PHQ-9 and GAD-7 scores was consistently small, with considerable overlap in the confidence intervals of the mean estimates in all analyses, and likely do not reflect clinically meaningful changes. Finally, we found that the effect of lockdowns on symptoms of depression and anxiety were largely unmodified by personality traits. Irrespective of time period, there was a trend for people who scored highly on neuroticism to report more depressive and anxiety symptoms, and conversely, those who self-reported high conscientiousness reported lower levels of these symptoms.

Secondary analysis of Victorian participants (the cohort that experienced the longest and strictest lockdowns in Australia, and arguably, the world) indicated that mental health generally improved across all groups, with no change in trajectory based on any of the potential modifying factors. People with a history of medical conditions or clinically diagnosed mental health problems, or younger participants (i.e. aged below 65 or 65–70 with no medical conditions), displayed greater depressive and anxious symptoms on average (consistent with the research by Van Rheenen et al^27^). Additionally, people with caring responsibilities showed more anxious symptoms compared with those with no caring responsibilities. These results are consistent with other studies; despite greater risks and potential difficulties faced by older people during the pandemic and associated restrictions, this age group has been found to report lower rates of psychological distress and poor mental health and lower scores on mood symptoms scales across multiple COVID-19 studies.^10,28,32^ However, findings from the secondary analyses may reflect characteristics of these particular groups, independent of a pandemic. For example, research conducted before the pandemic indicated that depressive or anxiety symptoms tended to decrease with increasing age.^33^ Additionally, the negative effects of caring on psychological health have been well established, including on anxiety, depression and burden.^29^ and an Australian study found that carers with unmet support needs faced a two-fold increase in the odds of psychological distress relative to those with no unmet needs.^34^

Our analysis is one of the few surveys in Australia that capture data leading into and following the second wave and the infamously strict restrictions imposed during that time. There are limited studies published to date with longitudinal data for mental health and well-being on people living in Australia during the pandemic, only one of which reported data collected before the second wave.^10,14,15^ Research conducted in Australia before the pandemic reported lower PHQ-9 scores (means ranging from 3.71 to 5.7)^35,36^ than the pre-lockdown mean reported for this study (mean 6.26, s.d. 5.24), which suggests a general increase in depressive symptoms triggered by the pandemic. However, consistent with our results of improved mental health scores across the three time periods, the UCL COVID-19 social study also found improving mental health over time,^22^ and findings from China indicated that rates of psychological distress declined in the weeks following the initial outbreak.^37^ Other findings from Australia also showed that people who were still working early in the pandemic showed improved mental health over time.^11^ This may reflect some adjustment to a ‘new normal’ for individuals whose lives and livelihoods were not severely affected by the pandemic, or a gradual return to the pre-pandemic baseline after the initial outbreak.

In contrast, two cross-sectional surveys administered in April 2020 and July to August 2020 found substantially greater prevalence of clinically depressive and generalised anxiety symptoms in Victoria compared with other states and territories during the second wave.^38^ Another longitudinal survey, conducted between April and September 2020, found increased depressive symptoms and poorer self-reported coping, hopefulness and quality of life during the second wave of the pandemic, compared with the first wave.^15^ Hopefulness, however, rebounded in September 2020 across their sample, likely because of to the impending easing of government-imposed restrictions. Anxiety, stress and resilience, on the other hand, remained relatively stable. Other research has consistently shown that mental health and psychological well-being have been adversely affected during the pandemic by job loss or reduced work, financial stress, pre-existing mental health disorders, feeling that government restrictions were negatively affecting their daily lives or that they would continue for the long term, loss of social connectedness, or worry about contracting COVID-19.^7,8,10,15,27^ The analyses presented in this study controlled for as many of those factors as the available data would allow, to examine the effect of pandemic-associated restrictions on mental health. Taken together, the present results and other findings in the literature suggest that individual experiences of the pandemic and resultant effects on mental health are driven by the ways in which public health measures and restrictions have directly affected the lives of people rather than being in lockdown *per se*. It is, therefore, necessary to examine individual factors that may influence the way in which lockdown may affect participants’ mental health.

### Limitations

Although significant in our model, the point estimates or the limits of the confidence intervals did not reflect clinically meaningful differences for either depression or anxiety symptoms (overall, or by location of residence). Although the PHQ-9 and GAD-7 are well-validated scales for the purpose of assessing depression or anxiety symptoms, it is important to note that these measures were collected via self-report and are not diagnostic data, and as brief self-report instruments, are not as valid as more detailed measures. There are also some self-selection biases to consider. Convenience sampling likely resulted in sampling bias. Most participants were women (80%), very few opted for the option of hard-copy surveys and the survey was predominately conducted online, which may have skewed the sample of participants toward people who were more able and willing to access the necessary technology. Additionally, nearly 50% of the sample were in the most advantageous socioeconomic quintile and were in current employment, which skewed our sample as those in more socioeconomically disadvantaged and diverse circumstance experienced disproportionately higher mortality and morbidity associated with the virus,^39^ which would have affected mental health. Caution should therefore be taken in generalising our results to the wider population. Participants could also both join and leave the study at different time points, meaning that participants that contributed data during the pre-lockdown period may differ to participants that contributed data during the lockdown or post-lockdown periods. This means that differences observed could be attributable to differences between participants rather than within-individual improvements in mental health. Thus, improvements in mental health over time may not be a result of within-individual changes in mental health but could be explained by the number of participants who have provided outcome data at the different lockdown stages. However, our sensitivity analyses of participants that provided data across all time periods provided consistent findings (see Supplementary Table 10).

In summary, this study reports a lack of deterioration in mental health over time in people living in Australia during the first year of the COVID-19 pandemic, even throughout notoriously strict lockdowns and restrictions. These results are meaningful as there are limited longitudinal survey studies on the well-being effects of COVID-19, especially those that consider the ways in which public health measures and restrictions directly affect the mental health of people. Our findings reveal the cohorts that appear to be at greater risk of poor mental health and should be targeted for mental health supports and interventions. These were people with a history of clinically diagnosed mental health problems, younger participants (i.e. aged below 65, or below 70 if they had no medical conditions), people with a history of medical conditions, people with caring responsibilities and people with more neurotic personality traits. For example, given that high conscientiousness was associated with better mental health in our study, targeted mental health supports could focus on improving conscientiousness in at-risk groups. This also suggests that public health messaging highlighting social responsibility may resonate with, and thus support the mental health of, highly conscientious people during a pandemic; however, further research is required to investigate this. Other future research should examine the short- and long-term effects of job loss, financial stress and social isolation on mental health, to better understand differential effects of lockdown consequences on mental health trajectories across the pandemic.

These results provide insight into how people are navigating the pandemic and tracking mental health across time. This may help researchers, clinicians and policy makers to better understand the effects of COVID-19 and restriction measures on individuals, identify which groups are most at risk, and inform the advice that people are given about how to maintain mental well-being and mental health during a pandemic. As the pandemic and its extended effects continue, investigations such as this study into the impact of the COVID-19 pandemic on mental health are vital for informing service delivery and supports and providing more evidence, as future strict public health measures such as lockdowns are considered for pandemics or other public health reasons.

## Data Availability

The data that support the findings of this study are available from the corresponding author, A.M.Y.G., upon reasonable request.

## Supplementary material

For supplementary material accompanying this paper visit http://doi.org/10.1192/bjo.2023.65. click here to view supplementary material
