# Supplementary material for: The impact of strict lockdowns on the mental health and well-being of people living in Australia during the first year of the COVID-19 pandemic
Source: BJPsych Open. 2023 May 24;9(3):e90. doi: 10.1192/bjo.2023.65 (PMC10228230; doi:10.1192/bjo.2023.65)
Supplement: Supplementary file 1 [file S2056472423000650sup001.docx]

# Supplementary Material

Table S1: Missing data frequencies and proportions in the key variables of interest for individuals with incomplete survey status (n=295)

| **Variable** | **Frequency (%)** |
| --- | --- |
| PHQ | 295 (100%) |
| GAD | 295 (100%) |
| Postcode | 290 (98.3%) |
| Age | 276 (93.6%) |
| Gender | 273 (92.5%) |
| Caring responsibilities | 292 (99.0%) |
| History of mental health | 292 (99.0%) |
| Country of birth | 275 (93.2%) |

**Table S2:** number of repeated outcome measures provided by individuals with missing postcodes (n=44)

| ID | No of PHQ measures | No of GAD measures |
| --- | --- | --- |
| 27 | 0 | 0 |
| 34 | 0 | 0 |
| 39 | 2 | 2 |
| 64 | 0 | 0 |
| 112 | 2 | 2 |
| 307 | 0 | 0 |
| 326 | 0 | 0 |
| 407 | 1 | 1 |
| 437 | 0 | 0 |
| 439 | 2 | 2 |
| 441 | 0 | 0 |
| 504 | 1 | 1 |
| 519 | 1 | 1 |
| 528 | 1 | 1 |
| 580 | 19 | 18 |
| 619 | 21 | 21 |
| 625 | 1 | 1 |
| 629 | 0 | 0 |
| 676 | 3 | 3 |
| 757 | 4 | 4 |
| 772 | 0 | 0 |
| 790 | 1 | 1 |
| 798 | 0 | 0 |
| 818 | 0 | 0 |
| 832 | 4 | 4 |
| 853 | 0 | 0 |
| 882 | 0 | 0 |
| 920 | 0 | 0 |
| 944 | 0 | 0 |
| 965 | 0 | 0 |
| 983 | 0 | 0 |
| 1005 | 0 | 0 |
| 1007 | 1 | 2 |
| 1029 | 0 | 0 |
| 1052 | 0 | 0 |
| 1064 | 1 | 1 |
| 1076 | 16 | 16 |
| 1079 | 4 | 4 |
| 1091 | 0 | 0 |
| 1102 | 0 | 0 |
| 1133 | 16 | 16 |
| 1143 | 0 | 0 |
| 1149 | 0 | 0 |
| 1221 | 1 | 0 |

Note: The postcodes from individuals who have provided no responses for each outcome will not be included in the analysis; therefore, recovering their postcodes are not useful.

**Table S3.** Dates of the pre-major lockdown period for Victorian postcodes.

| **Pre-major lockdown period** | **Victorian postcodes** |
| --- | --- |
| 27^th^ May – 1^st^ July 2020 | 3012, 3021, 3032, 3038, 3042, 3047, 3055, 3060, 3064 |
| 27^th^ May – 4^th^ July 2020 | 3031, 3051 |
| 27^th^ May – 9^th^ July 2020 | 3753, 3658, 3762, 3764, 3444, 3522, 3758, 3523, 3660, 3435, 3662, 3521, 3659, 3756 |
| 27^th^ May – 2^nd^ August 2020 | All remaining Victorian postcodes |

**Table S4**: Descriptive characteristics of the complete case sample* and omitted participants from primary analysis with outcome measured by PHQ, at point at which they joined the study

|  |  | **Complete case sample** | | **Omitted participants** | |
| --- | --- | --- | --- | --- | --- |
|  |  | **N=767** | | **N=108** | |
|  |  | **n (%) or mean (SD)** | | **n (%) or mean (SD)** | |
| **Age** | years | 59.3 (16.5) (n=767) | | 60.2 (16.4) (n=102) | |
| **Gender** | Female | 622 | (81.10%) | 71 | (73.96%) |
|  | Male | 141 | (18.38%) | 24 | (25.00%) |
|  | Other / prefer not to say | 4 | (0.52%) | 1 | (1.04%) |
| **Employment status** | No | 340 | (44.33%) | 53 | (51.96%) |
|  | Yes | 427 | (55.67%) | 49 | (48.04%) |
| **Carer responsibilities** | No | 604 | (78.75%) | 56 | (73.68%) |
|  | Yes | 163 | (21.25%) | 20 | (26.32%) |
| **History of clinically diagnosed mental health problems** | No | 618 | (80.57%) | 70 | (75.27%) |
|  | Yes | 149 | (19.43%) | 23 | (24.73%) |
| **Area of residence affected by the metropolitan VIC lockdown** | Metro Victoria | 483 | (62.97%) | 64 | (59.26%) |
|  | Non-Victoria | 178 | (23.21%) | 28 | (25.93%) |
|  | Regional Victoria | 106 | (13.82%) | 16 | (14.81%) |
| **SEIFA IRSAD quintile** | quintile 1 | 58 | (7.56%) | 4 | (3.70%) |
|  | quintile 2 | 64 | (8.34%) | 12 | (11.11%) |
|  | quintile 3 | 113 | (14.73%) | 18 | (16.67%) |
|  | quintile 4 | 172 | (22.43%) | 30 | (27.78%) |
|  | quintile 5 | 360 | (46.94%) | 44 | (40.74%) |
| **Born in or outside Australia** | Australia | 557 | (72.62%) | 74 | (70.48%) |
|  | Other | 210 | (27.38%) | 31 | (29.52%) |

* Complete case analysis omits participants with missing data on key covariates (n=53), as well as those who responded only once during the course of the survey and had a missing value for PHQ (n=54).

**Table S5**: Descriptive characteristics of the complete case sample* and omitted participants from primary analysis with outcome measured by GAD, at point at which they joined the study

|  |  | **Complete case sample** | | **Omitted participants** | |
| --- | --- | --- | --- | --- | --- |
|  |  | **N=764** | | **N=111** | |
|  |  | **n (%) or mean (SD)** | | **n (%) or mean (SD)** | |
| **Age** | years | 59.4 (16.4) (n=764) | | 59.3 (16.8) (n=105) | |
| **Gender** | Female | 680 | (80.86%) | 72 | (72.73%) |
|  | Male | 156 | (18.55%) | 26 | (26.26%) |
|  | Other / prefer not to say | 5 | (0.59%) | 1 | (1.01%) |
| **Employment status** | No | 377 | (44.56%) | 54 | (51.43%) |
|  | Yes | 469 | (55.44%) | 51 | (48.57%) |
| **Carer responsibilities** | No | 582 | (70.98%) | 60 | (75.95%) |
|  | Yes | 238 | (29.02%) | 19 | (24.05%) |
| **History of clinically diagnosed mental health problems** | No | 669 | (79.93%) | 73 | (76.04%) |
|  | Yes | 168 | (20.07%) | 23 | (23.96%) |
| **Area of residence affected by the metropolitan VIC lockdown** | Metro Victoria | 529 | (62.09%) | 63 | (56.76%) |
|  | Non-Victoria | 204 | (23.94%) | 31 | (27.93%) |
|  | Regional Victoria | 119 | (13.97%) | 17 | (15.32%) |
| **SEIFA IRSAD quintile** | quintile 1 | 60 | (7.04%) | 4 | (3.60%) |
|  | quintile 2 | 76 | (8.92%) | 13 | (11.71%) |
|  | quintile 3 | 126 | (14.79%) | 17 | (15.32%) |
|  | quintile 4 | 197 | (23.12%) | 31 | (27.93%) |
|  | quintile 5 | 393 | (46.13%) | 46 | (41.44%) |
| **Born in or outside Australia** | Australia | 616 | (72.56%) | 77 | (71.30%) |
|  | Other | 233 | (27.44%) | 31 | (28.70%) |

* Complete case analysis omits participants with missing data on key covariates (n=53), well as those who responded only once during the course of the survey and had a missing value for GAD (n=57).

**Table S6:** Adjusted analysis for the change in MH (measured by PHQ summary score) at three different time-periods by the area of residence (n=767)

**Table S6:** Adjusted analysis for the change in MH (measured by PHQ summary score) at three different time-periods by the area of residence (n=767)

|  | **Coef** | **95% Conf Int** | **p-value** |
| --- | --- | --- | --- |
| **Lockdown Period** |  |  |  |
| Pre-lockdown | Ref. |  |  |
| Lockdown | -0.37 | (-0.69,-0.06) | 0.020 |
| Post-lockdown | -1.42 | (-1.78,-1.07) | <0.001 |
| **Area of Residence** |  |  |  |
| Victoria | Ref. |  |  |
| Non-Victoria | -0.52 | (-1.40, 0.35) | 0.240 |
| Regional Victoria | 0.42 | (-0.71, 1.56) | 0.465 |
| **Lockdown Period X Area of Residence** |  |  |  |
| Pre-lockdown X Victoria | Ref. |  |  |
| Lockdown X Non-Victoria | -0.10 | (-0.55, 0.36) | 0.674 |
| Lockdown X Regional Victoria | -0.54 | (-1.09, 0.01) | 0.055 |
| Post-lockdown X Non-Victoria | 0.51 | (-0.02, 1.04) | 0.060 |
| Post-lockdown X Regional Victoria | -0.29 | (-0.95, 0.36) | 0.382 |

*Estimates are adjusted for participant age, gender, socio-economic status measured by SEIFA IRSAD, caring responsibilities, history of previous mental health, born in or outside Australia and whether engaged in paid employment

**Table S7:** Adjusted analysis for the change in MH (measured by GAD summary score) at three different time-periods by the area of residence (n=764)

|  | **Coef** | **95% Conf Int** | **p-value** |
| --- | --- | --- | --- |
| **Lockdown Period** |  |  |  |
| Pre-lockdown | Ref. |  |  |
| Lockdown | -0.01 | (-0.31, 0.29) | 0.951 |
| Post-lockdown | -0.73 | (-1.07,-0.40) | <0.001 |
| **Area of Residence** |  |  |  |
| Victoria | Ref. |  |  |
| Non-Victoria | -0.19 | (-0.98, 0.59) | 0.631 |
| Regional Victoria | 0.66 | (-0.36, 1.68) | 0.204 |
| **Lockdown Period X Area of Residence** |  |  |  |
| Pre-lockdown X Victoria | Ref. |  |  |
| Lockdown X Non-Victoria | -0.43 | (-0.85, 0.00) | 0.051 |
| Lockdown X Regional Victoria | -0.99 | (-1.51,-0.48) | <0.001 |
| Post-lockdown X Non-Victoria | 0.25 | (-0.25, 0.75) | 0.324 |
| Post-lockdown X Regional Victoria | -0.81 | (-1.43,-0.19) | 0.010 |

*Estimates are adjusted for participant age, gender, socio-economic status measured by SEIFA IRSAD, caring responsibilities, history of previous mental health, born in or outside Australia and whether engaged in paid employment

Table S8: Adjusted analysis for the change in MH (measured by PHQ and GAD summary scores)at three different time-periods by a) age group, b) caring responsibilities, c) history of clinically diagnosed mental health problems, d) medical history/co-morbidities, e) country of birth, f) keyworker and, g) children in household from LMMs adjusted for potential confounders

|  |  | **PHQ** | | | **GAD** | | |
| --- | --- | --- | --- | --- | --- | --- | --- |
|  |  | **Coef** | **95% Conf Int** | **p-value** | **Coef** | **95% Conf Int** | **p-value** |
| a) | **Lockdown Period** |  |  |  |  |  |  |
|  | Pre-lockdown | Ref. |  |  | Ref. |  |  |
|  | Lockdown | -0.15 | (-0.70, 0.40) | 0.589 | -0.15 | (-0.67, 0.36) | 0.562 |
|  | Post-lockdown | -0.91 | (-1.50,-0.33) | 0.002 | -0.61 | (-1.16,-0.06) | 0.030 |
|  | **At risk due to older age** |  |  |  |  |  |  |
|  | Yes | Ref. |  |  | Ref. |  |  |
|  | No | 2.83 | ( 1.66, 4.01) | <0.001 | 2.69 | ( 1.62, 3.76) | <0.001 |
|  | **Lockdown Period X At risk due to older age** |  |  |  |  |  |  |
|  | Pre-lockdown X Yes | Ref. |  |  | Ref. |  |  |
|  | Lockdown X No | -0.49 | (-1.12, 0.13) | 0.120 | -0.21 | (-0.80, 0.37) | 0.475 |
|  | Post-lockdown X No | -0.99 | (-1.67,-0.30) | 0.005 | -0.70 | (-1.34,-0.06) | 0.033 |
|  |  |  |  |  |  |  |  |
| b) | **Lockdown Period** |  |  |  |  |  |  |
|  | Pre-lockdown | Ref. |  |  | Ref. |  |  |
|  | Lockdown | -0.55 | (-0.84,-0.26) | <0.001 | -0.29 | (-0.56,-0.02) | 0.038 |
|  | Post-lockdown | -1.55 | (-1.88,-1.22) | <0.001 | -0.96 | (-1.28,-0.65) | <0.001 |
|  | **Caring responsibilities** |  |  |  |  |  |  |
|  | No | Ref. |  |  | Ref. |  |  |
|  | Yes | 1.01 | (-0.07, 2.09) | 0.068 | 1.40 | ( 0.42, 2.38) | 0.005 |
|  | **Lockdown Period X Caring responsibilities** |  |  |  |  |  |  |
|  | Pre-lockdown X No | Ref. |  |  | Ref. |  |  |
|  | Lockdown X Yes | -0.01 | (-0.66, 0.65) | 0.988 | -0.20 | (-0.81, 0.41) | 0.522 |
|  | Post-lockdown X Yes | -0.05 | (-0.79, 0.70) | 0.898 | -0.33 | (-1.03, 0.36) | 0.348 |
|  |  |  |  |  |  |  |  |
| c) | **Lockdown Period** |  |  |  |  |  |  |
|  | Pre-lockdown | Ref. |  |  | Ref. |  |  |
|  | Lockdown | -0.35 | (-0.64,-0.06) | 0.018 | 0.00 | (-0.27, 0.28) | 0.977 |
|  | Post-lockdown | -1.31 | (-1.64,-0.98) | <0.001 | -0.65 | (-0.96,-0.34) | <0.001 |
|  | **History of mental health issues** |  |  |  |  |  |  |
|  | No | Ref. |  |  | Ref. |  |  |
|  | Yes | 4.96 | ( 3.85, 6.06) | <0.001 | 4.24 | ( 3.24, 5.25) | <0.001 |
|  | **Lockdown Period X Previous mental health issues** |  |  |  |  |  |  |
|  | Pre-lockdown X No | Ref. |  |  | Ref. |  |  |
|  | Lockdown X Yes | -0.95 | (-1.60,-0.30) | 0.004 | -1.56 | (-2.15,-0.96) | <0.001 |
|  | Post-lockdown X Yes | -1.34 | (-2.11,-0.58) | 0.001 | -1.87 | (-2.58,-1.16) | <0.001 |
|  |  |  |  |  |  |  |  |
| d) | **Lockdown Period** |  |  |  |  |  |  |
|  | Pre-lockdown | Ref. |  |  | Ref. |  |  |
|  | Lockdown | -0.80 | (-1.15,-0.44) | <0.001 | -0.73 | (-1.06,-0.40) | <0.001 |
|  | Post-lockdown | -1.72 | (-2.11,-1.32) | <0.001 | -1.22 | (-1.60,-0.85) | <0.001 |
|  | **History of medical conditions** |  |  |  |  |  |  |
|  | Yes | Ref. |  |  | Ref. |  |  |
|  | No | -2.18 | (-3.12,-1.24) | <0.001 | -1.71 | (-2.56,-0.86) | <0.001 |
|  | **Lockdown Period X Previous medical conditions** |  |  |  |  |  |  |
|  | Pre-lockdown X yes | Ref. |  |  | Ref. |  |  |
|  | Lockdown X No | 0.57 | ( 0.04, 1.09) | 0.034 | 0.90 | ( 0.41, 1.39) | <0.001 |
|  | Post-lockdown X No | 0.33 | (-0.27, 0.93) | 0.277 | 0.39 | (-0.17, 0.95) | 0.174 |
|  |  |  |  |  |  |  |  |
| e) | **Lockdown Period** |  |  |  |  |  |  |
|  | Pre-lockdown | Ref. |  |  | Ref. |  |  |
|  | Lockdown | -0.85 | (-1.15,-0.55) | <0.001 | -0.58 | (-0.86,-0.30) | <0.001 |
|  | Post-lockdown | -1.84 | (-2.19,-1.49) | <0.001 | -1.30 | (-1.62,-0.97) | <0.001 |
|  | **Country of birth** |  |  |  |  |  |  |
|  | In Australia | Ref. |  |  | Ref. |  |  |
|  | Outside Australia | -1.95 | (-2.91,-0.99) | <0.001 | -1.59 | (-2.47,-0.71) | <0.001 |
|  | **Lockdown Period X Country of birth** |  |  |  |  |  |  |
|  | Pre-lockdown X In Australia | Ref. |  |  | Ref. |  |  |
|  | Lockdown X Outside Australia | 1.13 | ( 0.54, 1.72) | <0.001 | 0.93 | ( 0.38, 1.48) | 0.001 |
|  | Post-lockdown X Outside Australia | 1.06 | ( 0.39, 1.73) | 0.002 | 0.99 | ( 0.37, 1.61) | 0.002 |
|  |  |  |  |  |  |  |  |
| f) | **Lockdown Period** |  |  |  |  |  |  |
|  | Pre-lockdown | Ref. |  |  | Ref. |  |  |
|  | Lockdown | -0.43 | (-0.97, 0.10) | 0.110 | 0.03 | (-0.49, 0.55) | 0.906 |
|  | Post-lockdown | -1.69 | (-2.32,-1.06) | <0.001 | -1.19 | (-1.80,-0.57) | <0.001 |
|  | **Key worker** |  |  |  |  |  |  |
|  | Yes | Ref. |  |  | Ref. |  |  |
|  | No | 0.76 | (-0.39, 1.91) | 0.194 | 0.73 | (-0.33, 1.79) | 0.177 |
|  | **Lockdown Period X Key worker** |  |  |  |  |  |  |
|  | Pre-lockdown X No | Ref. |  |  | Ref. |  |  |
|  | Lockdown X No | -0.35 | (-1.00, 0.31) | 0.299 | -0.64 | (-1.27,-0.00) | 0.048 |
|  | Post-lockdown X No | -0.40 | (-1.17, 0.38) | 0.315 | -0.19 | (-0.94, 0.56) | 0.621 |
|  |  |  |  |  |  |  |  |
| g) | **Lockdown Period** |  |  |  |  |  |  |
|  | Pre-lockdown | Ref. |  |  | Ref. |  |  |
|  | Lockdown | -0.09 | (-0.67, 0.49) | 0.762 | -0.08 | (-0.62, 0.47) | 0.786 |
|  | Post-lockdown | -2.36 | (-3.10,-1.62) | <0.001 | -1.47 | (-2.16,-0.78) | <0.001 |
|  | **Children in household** |  |  |  |  |  |  |
|  | Yes | Ref. |  |  | Ref. |  |  |
|  | No | 0.56 | (-0.62, 1.74) | 0.354 | 0.12 | (-0.95, 1.18) | 0.831 |
|  | **Lockdown Period X Children in household** |  |  |  |  |  |  |
|  | Pre-lockdown X No | Ref. |  |  | Ref. |  |  |
|  | Lockdown X No | -0.57 | (-1.22, 0.09) | 0.088 | -0.34 | (-0.95, 0.27) | 0.278 |
|  | Post-lockdown X No | 0.85 | ( 0.04, 1.66) | 0.040 | 0.44 | (-0.32, 1.20) | 0.254 |
|  |  |  |  |  |  |  |  |

Table S9: Adjusted analysis for the change in MH (measured by PHQ and GAD summary scores)at three different time-periods by personality traits of a) extroversion, b) agreeableness, c) openness, d) conscientiousness, and e) neuroticism from LMMs adjusted for potential confounders.

|  |  | **PHQ** | | | **GAD** | | |
| --- | --- | --- | --- | --- | --- | --- | --- |
|  |  | **Coef** | **95% Conf Int** | **p-value** | **Coef** | **95% Conf Int** | **p-value** |
| a) | **Lockdown Period** |  |  |  |  |  |  |
|  | Pre-lockdown | Ref. |  |  | Ref. |  |  |
|  | Lockdown | -0.90 | (-1.75,-0.06) | 0.036 | -0.05 | (-0.85, 0.74) | 0.893 |
|  | Post-lockdown | -1.37 | (-2.32,-0.41) | 0.005 | -0.48 | (-1.38, 0.43) | 0.302 |
|  |  |  |  |  |  |  |  |
|  | **Personality trait-extroversion** | -0.05 | (-0.15, 0.05) | 0.330 | 0.04 | (-0.05, 0.13) | 0.421 |
|  |  |  |  |  |  |  |  |
|  | **Lockdown Period X Personality trait-extroversion** |  |  |  |  |  |  |
|  | Lockdown | 0.03 | (-0.04, 0.09) | 0.390 | -0.02 | (-0.08, 0.04) | 0.521 |
|  | Post-lockdown | -0.02 | (-0.09, 0.06) | 0.656 | -0.04 | (-0.11, 0.03) | 0.221 |
|  |  |  |  |  |  |  |  |
| b) | **Lockdown Period** |  |  |  |  |  |  |
|  | Pre-lockdown | Ref. |  |  | Ref. |  |  |
|  | Lockdown | -3.80 | (-5.20,-2.40) | <0.01 | -2.71 | (-4.03,-1.38) | <0.01 |
|  | Post-lockdown | -4.82 | (-6.39,-3.25) | <0.01 | -2.87 | (-4.36,-1.38) | <0.01 |
|  |  |  |  |  |  |  |  |
|  | **Personality trait-agreeableness** | -0.35 | (-0.50,-0.20) | <0.01 | -0.29 | (-0.43,-0.15) | <0.01 |
|  |  |  |  |  |  |  |  |
|  | **Lockdown Period X Personality trait-agreeableness** |  |  |  |  |  |  |
|  | Lockdown | 0.21 | ( 0.12, 0.30) | <0.01 | 0.15 | ( 0.07, 0.24) | <0.01 |
|  | Post-lockdown | 0.21 | ( 0.11, 0.31) | <0.01 | 0.12 | ( 0.02, 0.21) | 0.014 |
|  |  |  |  |  |  |  |  |
| c) | **Lockdown Period** |  |  |  |  |  |  |
|  | Pre-lockdown | Ref. |  |  | Ref. |  |  |
|  | Lockdown | 0.33 | (-0.88, 1.55) | 0.591 | -0.40 | (-1.53, 0.74) | 0.493 |
|  | Post-lockdown | -0.34 | (-1.74, 1.05) | 0.628 | -0.77 | (-2.07, 0.53) | 0.246 |
|  |  |  |  |  |  |  |  |
|  | **Personality trait-openness** | 0.04 | (-0.09, 0.17) | 0.550 | 0.04 | (-0.08, 0.16) | 0.540 |
|  |  |  |  |  |  |  |  |
|  | **Lockdown Period X Personality trait-openness** |  |  |  |  |  |  |
|  | Lockdown | -0.06 | (-0.13, 0.02) | 0.144 | 0.01 | (-0.06, 0.08) | 0.868 |
|  | Post-lockdown | -0.08 | (-0.17, 0.01) | 0.080 | -0.02 | (-0.10, 0.07) | 0.693 |
|  |  |  |  |  |  |  |  |
| d) | **Lockdown Period** |  |  |  |  |  |  |
|  | Pre-lockdown | Ref. |  |  | Ref. |  |  |
|  | Lockdown | -4.24 | (-5.66,-2.83) | <0.01 | -3.34 | (-4.65,-2.03) | <0.01 |
|  | Post-lockdown | -5.41 | (-7.01,-3.82) | <0.01 | -3.12 | (-4.61,-1.63) | <0.01 |
|  |  |  |  |  |  |  |  |
|  | **Personality trait-conscientiousness** | -0.52 | (-0.67,-0.38) | <0.01 | -0.31 | (-0.44,-0.18) | <0.01 |
|  |  |  |  |  |  |  |  |
|  | **Lockdown Period X Personality trait-conscientiousness** |  |  |  |  |  |  |
|  | Lockdown | 0.23 | ( 0.15, 0.32) | <0.01 | 0.19 | ( 0.11, 0.27) | <0.01 |
|  | Post-lockdown | 0.24 | ( 0.15, 0.34) | <0.01 | 0.13 | ( 0.04, 0.22) | 0.004 |
|  |  |  |  |  |  |  |  |
| e) | **Lockdown Period** |  |  |  |  |  |  |
|  | Pre-lockdown | Ref. |  |  | Ref. |  |  |
|  | Lockdown | 1.28 | ( 0.45, 2.12) | 0.003 | 1.33 | ( 0.55, 2.11) | 0.001 |
|  | Post-lockdown | 0.68 | (-0.23, 1.60) | 0.143 | 1.16 | ( 0.31, 2.02) | 0.008 |
|  |  |  |  |  |  |  |  |
|  | **Personality trait-neuroticisim** | 0.53 | ( 0.42, 0.65) | <0.01 | 0.58 | ( 0.48, 0.68) | <0.01 |
|  |  |  |  |  |  |  |  |
|  | **Lockdown Period X Personality trait-neuroticisim** |  |  |  |  |  |  |
|  | Lockdown | -0.17 | (-0.24,-0.10) | <0.01 | -0.15 | (-0.21,-0.08) | <0.01 |
|  | Post-lockdown | -0.20 | (-0.28,-0.13) | <0.01 | -0.20 | (-0.27,-0.13) | <0.01 |
|  |  |  |  |  |  |  |  |

Table S10- Mean PHQ and GAD scores by lockdown period for participants who attended the survey at all three lockdown periods (n=135)

| **Variable** | **Pre-lockdown** | | **Lockdown** | | **Post-lockdown** | |
| --- | --- | --- | --- | --- | --- | --- |
|  | **No of repeated measures** | **Mean** | **No of repeated measures** | **Mean** | **No of repeated measures** | **Mean** |
| PHQ | 342 | 5.37 | 1658 | 4.68 | 633 | 4.00 |
| GAD | 341 | 4.47 | 1659 | 4.00 | 644 | 3.74 |
